# Successful Control of Disseminated Intravascular Coagulation by Recombinant Thrombomodulin during Arsenic Trioxide Treatment in Relapsed Patient with Acute Promyelocytic Leukemia

**DOI:** 10.1155/2012/908196

**Published:** 2012-09-12

**Authors:** Motohiro Shindo, Katsuya Ikuta, Lynda Addo, Satoshi Ito, Mikihiro Fujiya, Yoshihiro Torimoto, Yutaka Kohgo

**Affiliations:** ^1^Division of Gastroenterology and Hematology/Oncology, Department of Medicine, Asahikawa Medical University, Asahikawa 078-8510, Japan; ^2^Oncology Center, Asahikawa Medical University Hospital, Asahikawa 078-8510, Japan

## Abstract

Disseminated intravascular coagulation (DIC) frequently occurs in patients with acute promyelocytic leukemia (APL). With the induction of therapy in APL using all-trans retinoic acid (ATRA), DIC can be controlled in most cases as ATRA usually shows immediate improvement of the APL. However, arsenic trioxide (ATO) which has been used for the treatment of relapse in APL patients has shown to take time to suppress APL cells, therefore the control of DIC in APL with ATO treatment is a major problem. Recently, the recombinant soluble thrombomodulin fragment has received a lot of attention as the novel drug for the treatment of DIC with high efficacy. Here, we present a relapsed patient with APL in whom DIC was successfully and safely controlled by rTM during treatment with ATO.

## 1. Brief Communication

Disseminated intravascular coagulation (DIC) frequently occurs in patients with acute promyelocytic leukemia (APL), contributing to bleeding complications, especially during treatment. With the induction of therapy in APL using all-trans retinoic acid (ATRA), DIC can be controlled in most cases as ATRA usually shows immediate improvement of the APL. However, arsenic trioxide (ATO) which has recently been used for the treatment of relapse in APL patients has shown to take time to suppress APL cells, therefore the control of DIC in APL with ATO treatment is a major problem. Recently, the recombinant soluble thrombomodulin fragment (rTM, Recomodulin; Asahi Kasei Pharm, Tokyo, Japan) has received a lot of attention as the novel drug for the treatment of DIC with high efficacy [1]. DIC may however occur as a result of various diseases, such as severe infection and malignant tumors, therefore more clinical experiences using rTM for various situations are needed to know the precise indications of rTM. Here, we present a relapsed patient with APL in whom DIC was successfully and safely controlled by rTM during treatment with ATO.

**Figure 1:**
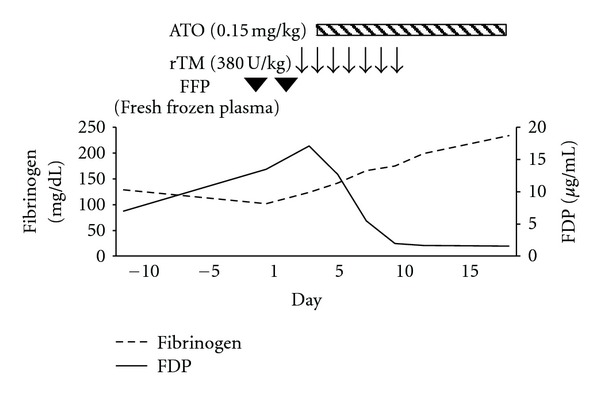
Clinical course of disseminated intravascular coagulation (DIC). The patient was admitted on day 1. The administration of recombinant thrombomodulin (rTM) was started on day 4, and then the administration of arsenic trioxide (ATO) was started on day 5. Fibrin/fibrinogen degradation product (FDP) levels decreased immediately, and fibrinogen levels gradually increased, indicating DIC was improved.

 A 38-year-old Japanese man was diagnosed with APL in 2007. At the time of first onset of the disease, no finding indicating DIC was observed. Chemotherapy combined with ATRA was performed as induction therapy. ATRA syndrome occurred, but that was successfully improved with the administration of steroid. After consolidation therapy, molecular CR was achieved. ATRA administration was continued intermittently for 2 years as maintenance therapy; this involved eight sequential courses of the daily administration of ATRA for 2 weeks followed by a 10-week follow-up period without the use of ATRA. In 2011, however, PML-RAR*α* fusion transcript was detected by reverse-transcriptase polymerase chain reaction in his bone marrow, indicating molecular relapse of APL. He was then readmitted to our hospital. Upon admission, the laboratory data showed that his WBC was 2.67 × 10^9^/L without any blasts. The hemoglobin concentration was 151 g/L, and the platelet count was 174 × 10^9^/L, both within normal range. Regarding coagulation tests, prothrombin time, activated partial thromboplastin time, and antithrombin III levels were all within normal limits. However, fibrinogen levels reduced to 102 mg/dL whereas fibrin/fibrinogen degradation product (FDP) levels increased to 13.5 *μ*g/mL. Plasmin-alpha 2 plasmin inhibitor complex (PIC) and thrombin-antithrombin complex (TAT) levels increased to 4.3 *μ*g/mL and 8.6 ng/mL, respectively. From the data obtained, DIC was diagnosed according to the diagnostic criteria established by the Japanese Ministry of Health and Welfare (JMHW DIC criteria) [2]. Supplementation with fresh frozen plasma and the intravenous administration of rTM at a dose of 380 unit/kg/day were started. ATO therapy was also initiated on the following day. The coagulation results were observed to have improved rapidly after the administration of rTM; rTM therapy was maintained for 7 days without any adverse effect (Figure 1). A second molecular CR was successfully achieved without the progression of DIC.

For the treatment of relapse in APL, ATO has in recent times proved useful with high efficacy; the effect of ATO treatment has however been observed to appear gradually. For instance, the median time to clinical CR after a single consolidation course of ATO was reported to be 59 days (range, 28 to 85 days) [3], whereas the median time needed to achieve CR by ATRA was reported to be 42 days (range, 14 to 98 days) [4]. This suggests that the control of DIC during the treatment of relapsed APL by ATO is a major problem that must be addressed. 

In the treatment of DIC, a novel drug which has shown high efficacy has recently been introduced. Thrombomodulin (TM) is an endothelial cell surface membrane-bound glycoprotein that forms a complex with thrombin. This complex activates protein C rapidly which then inactivates factors Va and VIIIa, resulting in the suppression of further thrombin generation. Soluble TM fragments have been produced as a recombinant protein that can bind thrombin and activate protein C, thereby resulting in the modulation of coagulation pathways just as membrane-bound TM, and that has been shown to improve DIC [1, 5] and other conditions such as veno-occlusive disease after haematopoietic stem cell transplantation [6]. In the present study, the growth of relapsed APL cells was found to be rapid and the DIC seemed to progress rapidly, and so we used rTM in the treatment. An immediate improvement in the coagulation markers was observed without any complications such as bleeding or thrombosis; the treatment of APL by ATO then proceeded successfully. Although further study with similar case studies is necessary, we suggest the possibility of rTM as a useful drug in the management of DIC during treatment with ATO in patients with relapsed APL.
